# A Pre-Post Intervention-Based Study Investigating the Impact of Standardized Parenteral Nutrition at Tertiary Neonatal Intensive Care Unit in Karachi, Pakistan

**DOI:** 10.7759/cureus.15226

**Published:** 2021-05-25

**Authors:** Vikram Kumar, Anum Rahim, Erum Choudry, Rafia Jabbar, Waqar H Khowaja, Shabina Ariff, Syed Rehan Ali

**Affiliations:** 1 Neonatology, Indus Hospital and Health Network, Karachi, PAK; 2 Indus Hospital Research Center, Indus Hospital and Health Network, Karachi, PAK; 3 Indus Hospital and Research Center, Indus Hospital and Health Network, Karachi, PAK; 4 Pediatrics, Indus Hospital and Health Network, Karachi, PAK; 5 Pediatrics and Child Health, Aga Khan University Hospital, Karachi, PAK

**Keywords:** neonatology, nicu, standardized parenteral nutrition, parenteral nutrition, individualized parenteral nutrition

## Abstract

Introduction

Conventionally, various parenteral nutrition (PN) components are individually administered considering an individual neonate's requirements. More recently, standardized PN (SPN) formulations have been initiated for preterm neonates, which may benefit from the enhanced nutrient supply, less administration and prescription errors, reduced risk of infectious disease, and cost-effectiveness.

Methodology

A multicentered, pre-post intervention-based study was conducted at tertiary neonatal intensive care units (NICUs) in Karachi, Pakistan. Post-graduate residents of neonatology and pediatrics working in NICUs were included in the study, and their perspective was attained regarding PN formulation and a prescription for time consumption, ease, calculation errors, and general feedback. Independent T-test was applied to assess the statistical difference between the pre-and post-implementation of PN formulation for total time required for PN calculation, whereas for the rest of the quantitative variables Mann-Whitney U test was computed.

Results

The total time required to do the entire writing process, calculating and ordering PN, was 17.1±6.9 whereas significantly (p-value of <0.0001) reduced to 10.5±5.7 after implementing SPN prescriptions. Calculation errors were reduced from 32% to 12%, and writing errors were also decreased from 35% to 8% when the standardized parenteral nutritional formulation was applied.

Conclusion

Our findings show that implementing standardized prescriptions in the NICU has improved medication safety, with the most consistent benefit by reducing medication errors and time management. The SPN prescriptions save time for post-graduate residents, physicians, and pharmacists by eliminating previously required repetitive activities and calculations.

## Introduction

Nutrition is necessary for all living things for routine needs of the body, development, and growth. Balanced nutrition in early life has a long-term impact on growth, development metabolism, and general health [1]. Prematurely born neonates are critical and not ready to suck, swallow, assimilate, and absorb their diet properly [2]. Their gastrointestinal tract is not mature enough to tolerate direct full feed, so they are fed on gradual enteral nutrition [3]. Due to limited energy supply through enteral nutrition, an alternative source of nutrition and energy parenteral nutrition (PN) is provided, which helps transition from minimal to full enteral nutrition to fulfill body requirements [4].

Parenteral nutrition is an essential part of the management of premature infants with very low birth weight (VLBW) admitted to neonatal intensive care units (NICUs). PN constitutes macronutrients, micronutrients, minerals, and trace elements; these are combined in different ways to provide adequate energy and optimal recommended daily intake (RDI) [5]. If appropriate nutrition is not provided as per RDI, it may lead to growth failure, affect the neurodevelopmental outcome, and cause morbidities [6].

Manufacturing an intravenous composition of PN needs multiple stable components of predefined concentration in the appropriate amount to ensure the optimal provision. The mixture must be composed under aseptic measures to avoid infection risks. Its complex nature, specifications, and appropriateness make it a high-risk therapy [7,8]. Due to the complex nature of PN composition, there are multiple chances of errors during its compounding that can compromise neonatal safety and health [9].

For better quality achievement and reduced errors, the American Society of Enteral and Parenteral Nutrition (A.S.P.E.N) published guidelines for parenteral nutrition ordering, order review, compounding, labeling, and dispensing [10]. Studies have reported that standardized parenteral nutrition (SPN) is relatively safe, cost-effective, and provides adequate nutrition with fewer chances of errors. It provides more calories than individualized parenteral nutrition (IPN) and increases amino acid, calcium, and phosphate intake while reducing early weight loss [11-13].

To determine the impact of SPN formulation and prescription for neonates at a tertiary NICU in Karachi, we adopted the Australian group consensus 2012 to SPN practices for neonates [14]. We implemented the SNP protocol for neonates in NICUs in two major tertiary care hospitals in the city, named Aga Khan University Hospital (AKUH) and Indus Hospital and Health Network (IHHN), Karachi, Pakistan. The main objective of this study was to assess the benefits of SPN in terms of calculation errors, prescription errors, and time management before and after implementation in the NICU.

## Materials and methods

Study setting and population

From June 2020 to September 2020, a pre-post intervention study was conducted at a tertiary NICU of the IHHN and AKUH, Karachi, Pakistan. Post-graduate residents of neonatology and pediatrics posted in the NICU were included in the study. After obtaining the ethical approval from the Institutional Review Board (IRD_IRB_2020_06_004), a survey was designed on REDCap to gather information regarding the level of trainees, their perspectives regarding PN formulation, general feedback, and difficulty level. The prescriptions were also assessed for time consumption, ease, and errors.

The project was initially implemented at AKUH, a Joint Commission International (JCI) accredited hospital in Karachi, Pakistan, and has a level 3 NICU comprising 24 beds. The annual birth rate is around 5000, out of which 16-17% are born prematurely. Annual NICU admissions are 1200-1300/year. Later, the protocol was replicated with success at IHHN, Karachi, which comprises a 12 bedded level 3 tertiary care NICU with 700-800 births/year.

Pre-intervention phase

Prescription and Calculation of PN 

In the pre-implementation phase, the assigned resident reviewed the need for PN with a team of attending physicians, followed by calculations for different constituents of PN, keeping in view the recommended dietary allowance (RDA) and the most recent biochemical profile of the admitted infant. After a thorough review of each calculation, a prescription request to the pharmacy was generated; this exercise had to be repeated daily. The concentration of all the ingredients had to be changed on different days depending upon the patient's daily fluid balance, most recent biochemical profile, and blood sugar status. This was followed by writing on the prescription paper for the nurses. *PN Pharmacy Review Process*

The pharmacist received PN requests through the hospital software; counterchecked each calculation while reviewing the biochemical profile of the infant via Patient Profile Viewer (PPV). If the pharmacist deemed any necessary modifications to be made, he communicated it with the concerned physician on the telephone. Subsequently, the request was then sent to the PN technician for formulation.

PN Reviewed by the Nurse

Once the PN bag arrived from the pharmacy, the inpatient nurse matched the label with the prescription before starting the nutrition through a parenteral route.

Post-intervention phase

Standardization of PN for Neonates

A standardized protocol for PN was implemented at the AKUH and IHHN after the approval from the hospital's nutrition committee, pharmacy, and drug committee. This protocol included three standards (starter, standard term, and standard preterm) and two optional (high sodium, low glucose) standards PN. Following were the important agenda points approved by the nutrition committee:

1. To standardize PN indication in NICU as every preterm, irrespective of gestational age, should receive PN.

2. To develop five different categories of PN.

*2.a. Three standards: *(i) Standard starter PN for the first 24 hours after birth and can be used later for babies with hyperkalemia or renal failure with fluid restriction. (ii) Standard preterm PN for preterm neonates after the first 24 hours of life. (iii) Standard term PN for term neonates after the first 24 hours of life.

*2.b. Two optional standards according to biochemical profiles of neonates: *(i) Standard high sodium PN for neonates with hyponatremia. (ii) Standard low glucose PN for neonates with hyperglycemia. (iii) Uniform standard of practice was established constituting the following points: (1) Indication criteria for PN administration (as illustrated in Table 1). (2) Ingredient and constituent for different categories of PN for pharmacists as a standard reference document shown in Table 2. (3) Provision of nutrients and electrolytes per kg per day at different rates of PN infusion for better understanding of physicians, residents, and nurses. (4) Biochemical and clinical conditions which warrant SPN, then the infant can be switched to IPN (Table 3). (5) A standard biochemical profile monitoring schedule was also developed as shown in Table 4.

**Table 1:** Indications for parenteral nutrition administration

Indications
Preterm birth (<32 weeks gestation)
Preterm baby with birth weight <1200 g
Malabsorption syndrome
Suspected/confirmed necrotizing enterocolitis
Any clinical condition which causes a delay in enteral feeding
Having evidence of perinatal asphyxia
Intrauterine growth retardation with absence or reversal of diastolic flow in the umbilical artery
Any neonate unable to achieve full feed by the fifth day of life

**Table 2:** Standardized parenteral nutrition: volumes and concentration in different categories *Only for a central line. **If electrolytes and sugars are normal. ***Consider if POCT blood sugar > 200, 3-4 episodes, confirmed by RBS. ****Consider serum sodium <135 ml, unable to receive oral sodium. Hypertonic saline/normal saline boluses should be avoided during high sodium PN, # can be given to patients with hyperkalemia and renal failure.

Constituent per 100 ml						
Ingredients with concentration	Age	At birth	After 24 hours of life**		Optional	
	Category	Starter	Standard preterm	Standard term	Low glucose***	high sodium****
Proteins (10% amino)		25 ml = 2.5 g/100 ml				
Dextrose percentage		8 g/100 ml	~8 g/100 ml	~10 g/100 ml	~7 g/100 ml	7.8 g/100 ml
25% dextrose		6 ml	12 ml	25.3 ml	6.3 ml	12 ml
10% D/W		65 ml	49.3 ml	36 ml	55 ml	48 ml
Lipid (20% IVFE)		0	9.5 ml (1.9 g/100 ml)			
MgSO_4_ (8 mEq/2 ml)		0.1 ml (0.4 mEq/100 ml)				
Potassium phosphorus (KPO) potassium (K) 1.5 mEq per 0.35 ml Phosphorus (Ph) 1 mmol per 0.35 ml		0	0.35 ml (K - 1.5 mEq/100 ml, Ph - 1 mmol/100 ml)			
NaCl 2.5 mEq/ml		1 ml (2.5 mEq)	1.2 ml (Nacl - 3 mEq/100 ml)			2.4 ml (6 mEq/100 ml)
Zinc (1 mg/ml)		0.3 ml - 300 µg per 100 ml				
Calcium gluconate (10%)		1.25 ml (125 mg/100 ml)				
Multivitamins (preferably both lipid and water-soluble as per concentration)		1 ml				
Heparin* (0.5 IU/ml)		0.1 ml (0.05 unit per 100 ml)				

**Table 3:** Consideration for individualized parenteral nutrition PN: parenteral nutrition, PNALD: parenteral nutrition-associated liver disease, GIR: glucose infusion rate, BUN: blood urea nitrogen, SBR: serum bilirubin, SGPT: serum glutamic-pyruvic transaminase.

Consider individualized PN/fluid electrolytes mixture, if a neonate has
Hypoglycemia despite adequate GIR through standard PN
Hypernatremia >150 mEq/dl despite adequate fluids
BUN > 50
Hypermagnesemia (>2.5 mEq/dl)
PNALD (SBR > 2 mg/dl, or direct bilirubin > 20% of total, SGPT > 50, Gamma GT > 100)
Hyperglycemia despite low glucose PN
Needs fluids through PN > 160 ml/kg/day

**Table 4:** Lab monitoring for biochemically and clinically stable infants CBC: complete blood count, BUN: blood urea nitrogen, LFTs: liver function tests, SBR: serum bilirubin.

Twice daily	Daily	Twice weekly (Monday, Thursday)	Weekly (Monday)
Electrolytes, BUN, creatinine, and other biochemical profile to be monitored twice daily if	Electrolytes first 72 hrs	After the first 72 hrs of neonates on PN	Liver function test
- Negative fluids balance (50 ml/kg/day)	Calcium, if <6.5 mg/dl	BUN, Cr	Phosphorus, calcium
Anuria (>12 hrs after the first 24 hrs)	Magnesium if < 1.4 mg/dl	CBC for the first week	Na, K once-off PN
Receiving high electrolytes fluids			CBC after stabilization
	SBR if clinically icteric and/or required phototherapy		

Data analysis

Data were analyzed using SPSS 26.0. Mean ± SD was calculated for total time taken for PN calculation, whereas median (IQR) was reported for the number of PN orders per day, number of PN calculations per prescription, calculations, writing, and software time in minutes, number of correction calls received from pharmacy and number of changes required to be done. Independent T-test was applied to assess the statistical difference between the pre-and post-implementation of PN formulation for the total time required for PN calculation, whereas the Mann-Whitney U test was applied for the rest of the quantitative variables. Level of residency training, calculation error, writing error, and suggested practices were presented with frequency and percentages, and Pearson's chi-square test was applied for assessing the differences. However, the Fisher exact test was used to determine the differences for software error and suggested practices. A P-value of <0.05 was considered significant.

## Results

This study was conducted in two phases: pre-implementation and post-implementation. In the first phase, 34 residents, and in the second phase, 48 residents participated in the study.

The PN calculation was required for each ingredient like macronutrients, micronutrients, electrolytes, mineral, fluid volume while adjusting the fluid volume in medication and feeds. Time consumed for all the calculations mentioned above was 10 minutes (5-12) in the pre-implementation phase and 1 minute (0.5-4) in the post-implementation phase with a significant difference (p-value <0.0001). The time needed in minutes for order writing in pre-implementation was five minutes (3-5), and post-implementation was one minute (0-2) with a p-value of <0.0001 that was significant, as shown in Table 5.

**Table 5:** Pre-and post-implementation assessment *P-value<0.05, **P-value<0.0001. T: independent sample T-test, M: Mann-Witney U test, P: Pearson Chi-square, f: Fisher's exact test.

Variables	Pre-implementation, n (%)	Post-implementation, n (%)	Combine, n (%)	P-value
Postgraduate residents	34	48	82	
Level of training				
R1	0	14 (29.2)	14 (17.10)	0.0000^**P^
R2	11 (32.4)	22 (45.8)	33 (40.2)	
R3	17 (50.0)	6 (12.5)	23 (28.0)	
R4	6 (17.6)	6 (12.5)	12 (14.6)	
PN prescription assessment				
Number of PN/day	4 (3-5)	3.5 (2-5)	4 (3-5)	0.014^*M^
Number of PN calculation/day	2.5 (1-5)	8 (2.5-10)	5 (1-9)	0.0000^**M^
Calculation time/mins	10 (5-12)	1 (0.5-4)	5 (1-10)	0.0000^**M^
Writing time/mins	5 (3-5)	1 (0-2)	2 (0.5-5)	0.0000^**M^
Software time/mins	2 (2-5)	1 (0.5-2.5)	2 (1-3)	0.0000^**M^
Total time/mins	17.1 (6.9)	10.5 (5.7)	13.3 (7.0)	0.0000^**T^
Number of correction calls	1.5 (1-3)	1 (1-2)	1 (1-2)	0.031^*M^
Number of changes	2 (1-2)	1 (1-1)	1 (1-2)	0.002^*M^
Calculation error				
Never	6 (17.6)	8 (16.7)	14 (17.1)	0.031^*P^
Occasional	17 (50.0)	34 (70.8)	51 (62.2)	
Frequent	11 (32.4)	6 (12.5)	17 (20.7)	
Writing error				
Never	11 (32.4)	10 (20.8)	21 (25.6)	0.0000^**P^
Occasional	11 (32.4)	19 (39.6)	30 (36.6)	
Frequent	12 (35.3)	4 (8.3)	16 (19.5)	
Not applicable	0	15 (31.3)	15 (18.3)	
Software error				
Never	10 (29.4)	3 (6.3)	13 (15.9)	0.003^*f^
Occasional	15 (44.1)	33 (68.8)	48 (58.5)	
Frequent	9 (26.5)	7 (14.6)	16 (19.5)	
Not applicable	0	5 (10.4)	5 (6.1)	
Level of difficulty in ordering PN				
Easy	12 (35.3)	35 (72.9)	47 (57.3)	0.001^*f^
Moderate	20 (58.8)	13 (27.1)	33 (40.2)	
Hard	2 (5.9)	0	2 (2.4)	
Practices				
Satisfied/continue	11 (22.9)	28 (82.4)	39 (47.6)	0.0000^**P^
Needs revision	37 (77.1)	6 (17.6)	43 (52.4)	

In the pre-implementation phase, the order requires the following three, i.e., concentration and amount of ingredients written on the residents order paper, entered in the pharmacy software template, and then manually written paper shared with pharmacy. In the post-implementation phase, the order requires only one step' in one unit (AKUH); the post-graduate trainee enters a fixed amount of each ingredient in Physician Order Entry (POE) software and in another unit (IHHN) pre-designed pneumonic to be selected in POE for different categories of standard PN. So, time consumption in post-implementation was one minute (0.5-2) than pre-implementation phase 2 (2-5).

The total time required to do the entire writing process, calculation, and ordering of PN was 17.1 ±6.9, whereas it significantly (p-value <0.0001) reduced to 10.5±5.7 after implementing PN formulation. Calculation Errors (32%) and writing errors (35%) were reduced up to 12% and 8%, respectively, in the pre-and post-implementation phase. In the post-implementation phase, order entry errors in POE Software decreased to 15% compared to frequent (26%) in the pre-implementation phase.

In the pre-implementation phase, the individualized (PN) satisfaction level was 22.9%, which was increased to 82% with SPN (p-value <0.0001). Similarly, a significant difference was observed in practices as the pharmacy's revision calls were reduced to 17.6% compared to 77.1% in the pre-implantation phase with a p-value of <0.0001. Total 59% of the residents reported difficulty level in ordering prescriptions for individualized PN as moderately hard, and for SPN, 73% of residents responded to it as easy. The entire process of requesting and formulating standard PN, overall, is much easier and less time-consuming when compared to IPN.

## Discussion

Pakistan has the third-highest number of neonatal deaths globally, with a reported neonatal mortality rate of 49 deaths per 1000 live births and an estimated 298,000 neonatal deaths yearly [15]. With poor maternal and neonatal health policies, incapacitated infrastructure, neonatal healthcare services are severely hampered in Pakistan. Neonatal nutrition services are limited to only a few tertiary care hospitals [16].

PN is an essential element in the care of neonates, particularly premature infants admitted to NICUs [17]. For VLBW babies, early "aggressive" parenteral feeding is now the recommended procedure. The aim of providing early PN to preterm infants is to improve mortality, morbidity, and long-term neurodevelopmental outcomes [18]. Since PN is an intravenous prescription; it is vulnerable to medication errors, where all the calculations are weight-based [19]. With over 50 constituents, PN is one of the most complex medications therapy. Prescription writing, transcription, calculation, and administration are all stages in the PN management process where errors can occur [20]. PN administration also has the potential to cause infectious complications as well as metabolic disturbances [21].

PN is administered under two regimes, the SPN and a much more precise and conventional IPN. IPN lets the physician prescribe a formulation tailor-made for a specific neonate, while SPN offers a more generalized solution to the issue. For neonates with VLBW who are also suffering from severe sickness or a metabolic disorder, IPN is the preferred formulation as it provides specific nutrients for the infant [22]. However, for infants merely needing a quick weight gain, several studies have suggested that SPN would be the superior choice as it promotes better absorption of glucose, amino acids along with calcium and phosphorus in the body [11,23]. Neonates in general have a weak immune system and are prone to infections, adding VLBW to the equation only aggravates the condition. SPN helps in decreasing the risk of infections more efficiently than IPN would [24]. PN itself is an expensive therapy, and it requires several additional resources like neonate monitoring, the cost of keeping the child in NICU, and hospital care. IPN further elevates the cost as more precision and care are needed to make the formulation. SPN offers a solution that is cheaper with relatively similar results to IPN for the infant’s diet. Studies suggest that SPN decreases the cost of PN therapy by 30-56% annually as there is no extra cost for materials, inventory-holding, and extra manpower to formulate the composition [11,25-27]. Therefore, SPN provides an advantage over IPN for the people of developing countries who can barely afford the cost for treatment of various neonatal health hazards much prevalent.

In many NICUs in Pakistan, PN is provided by individualized solutions prescribed and prepared for each infant rather than standardized stock solutions. This practice was a complex, time-consuming, brainstorming task for related healthcare workers. Therefore, in this study, we implemented the SPN ordering process in NICU to reduce physicians' workload and associated errors and delays. In support of our primary hypothesis, this study found that there was a significant difference observed in the calculation and writing errors (p-value <0.05 and <0.0001, respectively) after the standardization. Several studies have also shown that IPN is associated with increased medical errors, while SPN formulations have significantly fewer prescription errors in patients [27,28].

Each resident daily wrote around four to five prescriptions in the pre-implementation phase, which required approximately 30 minutes to calculate, prescribe, and request the pharmacy. The pharmacist involved in PN therapy is an essential part of the multidisciplinary nutrition management team [29]. The PN pharmacist required an almost similar time to review and recalculate the requested PN. This used to take around three to four hours, after which the PN technician starts PN formulation. The entire process takes up to six to eight hours altogether. After the implementation of SPN, the time required for ordering PN was also optimized. There was also a significant decrease in time consumption for each PN ordering process (p-value < 0.0001) that ultimately decreased the number of calls back for correction and errors (p-value <0.05). These results agree with the study highlighting the advantages of SPN for premature neonates [27].

Overall, this implementation was a rewarding experience that helped physicians, pediatrics and neonatology residents, pharmacists, nurses, and PN technicians. Now, residents and physicians can easily place requests for SPN for an infant according to their gestation, age, and biochemical status with volume rate per hour in pharmacy software without needing extensive unnecessary calculations. This strategy also forms part of an approach concerning quality control and good professional practice to prepare PN solutions in our study.

The limitation of this study includes a small sample size; however, we included all the available consenting post-graduate residents working in NICUs in both study centers. Due to the small sample size, advanced analysis was not performed; therefore, the effect of confounders was not adjusted. More data are required to assess the contrast of the impact of SPN over IPN on neonatal health and well-being by conducting large intervention-based studies or randomized control trials.

We propose to develop the required range of standardized neonatal PN formulations on a national level. To create the best possible formulations both nutritionally and pharmaceutically, there is a need to gather all available neonatologists, nutritionists, pediatric gastroenterologists, pharmacists, and nurses nationwide. The provision of sufficient nutrition without inducing biochemical derangement is an essential part of neonatal intensive care management. Concerning higher nutritional intake, weight gain, fewer medication errors, and cost of SPN may have advantages over IPN; without causing a significant biochemical disturbance. Therefore, we believe that SPN formulations would have an acceptable nutritional composition, resulting in satisfactory biochemical regulation and development in VLBW infants at various stages of their NICU stay [28].

## Conclusions

The implementation of SPN has revolutionized the overall standard of neonatal care. Our findings show that implementing standardized prescriptions in the NICU has improved medication safety, with the most consistent benefit by reducing medication errors and time management. We deduced that this standardization not only saved time but also decreased the workload on physicians, trainees, pharmacists, PN technicians, and nurses by eliminating previously required repetitive activities and calculations.
